# Differential Activation of Killer Cells in the Circulation and the Lung: A Study of Current Smoking Status and Chronic Obstructive Pulmonary Disease (COPD)

**DOI:** 10.1371/journal.pone.0058556

**Published:** 2013-03-11

**Authors:** Jia Wang, Richard A. Urbanowicz, Patrick J. Tighe, Ian Todd, Jonathan M. Corne, Lucy C. Fairclough

**Affiliations:** 1 COPD Research Group, The School of Molecular Medical Sciences, The University of Nottingham, Nottingham, Nottinghamshire, United Kingdom; 2 Department of Respiratory Medicine, Nottingham University Hospitals NHS Trust, Nottingham, Nottinghamshire, United Kingdom; University of London, St George's, United Kingdom

## Abstract

**Background:**

CD8^+^ T-lymphocytes, natural killer T-like cells (NKT-like cells, CD56^+^CD3^+^) and natural killer cells (NK cells, CD56^+^CD3^−^) are the three main classes of human killer cells and they are implicated in the pathogenesis of chronic obstructive pulmonary disease (COPD). Activation of these cells can initiate immune responses by virtue of their production of inflammatory cytokines and chemokines that cause lung tissue damage, mucus hypersecretion and emphysema. The objective of the current study was to investigate the activation levels of human killer cells in healthy non-smokers, healthy smokers, ex-smokers with COPD and current smokers with COPD, in both peripheral blood and induced sputum.

**Methods/Principal Findings:**

After informed consent, 124 participants were recruited into the study and peripheral blood or induced sputum was taken. The activation states and receptor expression of killer cells were measured by flow cytometry. In peripheral blood, current smokers, regardless of disease state, have the highest proportion of activated CD8^+^ T-lymphocytes, NKT-like cells and NK cells compared with ex-smokers with COPD and healthy non-smokers. Furthermore, CD8^+^ T-lymphocyte and NK cell activation is positively correlated with the number of cigarettes currently smoked. Conversely, in induced sputum, the proportion of activated killer cells was related to disease state rather than current smoking status, with current and ex-smokers with COPD having significantly higher rates of activation than healthy smokers and healthy non-smokers.

**Conclusions:**

A differential effect in systemic and lung activation of killer cells in COPD is evident. Systemic activation appears to be related to current smoking whereas lung activation is related to the presence or absence of COPD, irrespective of current smoking status. These findings suggest that modulating killer cell activation may be a new target for the treatment of COPD.

## Introduction

COPD is defined as a chronic inflammatory lung disease leading to irreversible airflow limitation as confirmed by spirometry [1]. Chronic bronchiolitis, emphysema and mucus hypersecretion are three main pathological features of COPD [2], [3], which result in the common and characteristic symptoms, including chronic cough and progressive dyspnoea [4]. COPD is predicted to be the third major worldwide cause of death by 2020 [5].

CD8^+^ T-lymphocytes, NKT-like cells and NK cells are the three main types of killer cells in the immune system and have been implicated in the pathogenesis of COPD [6], [7], [8], [9], [10], [11], [12]. Previous studies have shown that increased numbers of CD8^+^ T-lymphocytes are found in both the peripheral airways and lower respiratory tract in patients with COPD [13], [14]. We have previously shown that the numbers and cytotoxicity of NK and NKT-like cells are increased in induced sputum of patients with COPD [9] but are reduced in the peripheral blood [11], implying a potential role in disease pathogenesis.

For killer cells to be functional they need to be activated. CD69 and CD25 are cell surface markers of early and late stages of lymphocyte activation, respectively [15]. This activation involves several factors, such as interaction between antigen receptors and peptides presented by MHC class I molecules or MHC class I-related proteins. Human killer cells express immunoglobulin-like receptors (KIRs), belonging to the Ig superfamily (CD158 family), which bind to MHC Class I. They can be classified into two groups, namely, inhibitory KIRs and activating KIRs. Inhibitory KIRs possess long (L) cytoplasmic domains, such as KIR2DL or KIR3DL. Activating KIRs possess short cytoplasmic domains with an immunoreceptor tyrosine-based activation motif (ITAM)-bearing DAP12 adapter protein [16]. NKG2D is a unique activating receptor and a type II transmembrane-anchored glycoprotein. It transmits signals through its transmembrane segment associating with adaptor protein DAP10 and in the absence of DAP10, NKG2D is not able to be presented on the cell surface and is retained in the cytoplasm [17], [18]. Its expression can be regulated by cytokines; for example, IL-15 and TNF-α can enhance NKG2D expression whereas TGF-β causes its down-regulation [19], [20], [21]. In humans, KIR3DL1 (CD158e1) and NKG2D are two representative signalling receptors expressed by CD8^+^ T-lymphocytes, NK cells, NKT cells and γδ TCR^+^ T cells [18], [22], [23], [24], [25]. They can recognise MHC class I molecules and MHC class I-related proteins, respectively.

Several studies have investigated expression of activation markers on T-lymphocytes from peripheral blood, induced sputum and bronchial alveolar lavage (BAL) fluid from healthy smokers and COPD patients with varying results [26], [27], [28] but none have looked specifically at CD8 T lymphocytes, NKT-like and NK cells.

In this study, we investigated the activation levels (by measurement of CD69 and CD25 expression) of the three main classes of human killer cells, namely, CD8+ T-lymphocytes, NKT-like cells and NK cells, in peripheral blood and induced sputum. Samples were obtained from four groups of participants including healthy non-smoking volunteers, healthy smokers, ex-smokers with COPD and current smokers with COPD. To further detect the association between cell activation and their functional receptors in peripheral blood samples, we also examined the expression of the inhibitory receptor KIR3DL1 (CD158e1) and the activating receptor NKG2D in these four groups.

## Materials and Methods

### Study population and procedures

The Nottingham Research Ethics Committee approved the study protocol (REC reference 04/Q2403/102) and written informed consent was obtained from all participants before entering the study. Sixty-six participants were included in the peripheral blood activation study, thirty-two in the KIR study and twenty-six in the induced sputum study. Participants were diagnosed as having COPD according to the ATS guidelines; they were either current smokers with COPD or ex-smokers with COPD and had accrued at least a 20 pack year smoking history. Healthy smokers and healthy non-smokers, with an FEV1 above 80% of predicted, were matched, as far as possible, for age, with participants with COPD; healthy smokers were also matched for smoking history. Table 1 details the demographic and spirometric data of the participants in the activation study; table 2 the KIR study and table 3 the induced sputum study. Participants were excluded as previously stated [29], [30]; specifically, they were all non-atopic (negative skin prick test response to common allergen extracts including grass pollen, house dust mite, cat dander and dog hair) and all COPD patients had been free of an acute exacerbation of COPD for at least 6 weeks preceding the study. In addition, none had received antibiotics or corticosteroids (oral and inhaled) over the same period.

**Table 1:** Demographic and spirometric values of groups examined for the activation studies in the peripheral blood.

	Healthynon-smokers	Healthy smokers	exS-COPD	cuS-COPD
**Participants**	21	21	10	14
**Age (years)**	53 (42–68)	55 (43–68)	63 (49–75)	62 (45–77)
**Gender (M/F)**	14/7	10/11	5/5	7/7
**Packs/yrs**	0	43 (15–68)	45 (20–71)	53 (23–77)
**Smoking status (Current/Ex)**	0	21/0	0/10	14/0
**Chronic bronchitis** **(Yes/No)**	0	7/14	8/2	8/6
**FEV_1_ (% pred)**	107 (75–140)	102 (85–128)	50 (18–66)	41 (17–61)
**FEV_1_/FVC (%)**	90 (67–127)	80 (66–107)	51 (36–65)	46 (29–66)
**▵FEV_1_ post bronch**	2.4 (1.9–3.4)	2.7 (1.7–3.8)	1.4 (0.6–2.0)	1.9 (0.5–2.9)
**BMI (kg/m^2^)**	24.6 (17.8–32.2)	25.5 (19.2–36.9)	22.5 (20.6–24.7)	26.2 (18.3–35.6)
**Inhaled GCS (on/off)**	N/A	N/A	5/5	6/8
**MRC dyspnoea scale**	N/A	N/A	2 (1–4)	3 (2–4)
**Distance walked in 6 min (m)**	N/A	N/A	313 (140–492)	314 (50–431)
**BODE Index**	N/A	N/A	4 (1–5)	5 (2–7)

Results are expressed as median with range in brackets.

**Table 2:** Demographic and spirometric values of groups examined for the KIR studies in the peripheral blood.

	Healthynon-smokers	Healthy smokers	exS-COPD	cuS-COPD
**Participants**	8	8	7	9
**Age (years)**	54 (47–66)	53 (45–63)	62 (49–69)	61 (45–77)
**Gender (M/F)**	4/4	3/5	3/4	3/6
**Packs/yrs**	0	35 (32–40)	36 (20–51)	56 (30–77)
**Smoking status (Current/Ex)**	0	8/0	0/7	9/0
**Chronic bronchitis** **(Yes/No)**	0	3/5	5/2	6/3
**FEV_1_ (% pred)**	105 (75–126)	101 (85–114)	58 (52–66)	41 (30–61)
**FEV_1_/FVC (%)**	104 (67–120)	89 (66–101)	51 (36–65)	48 (29–53)
**▵FEV_1_ post bronch**	2.2 (1.9–3.0)	2.1 (1.7–2.9)	1.1 (0.6–1.5)	1.6 (1.1–2.4)
**BMI (kg/m^2^)**	22.8 (18.7–26.2)	24.7 (19.2–31.6)	21.5 (19.6–23.1)	23.2 (18.3–30.8)
**Inhaled GCS (on/off)**	N/A	N/A	4/3	3/6
**MRC dyspnoea scale**	N/A	N/A	2 (1–4)	3 (2–4)
**Distance walked in 6 min (m)**	N/A	N/A	277 (140–456)	308 (50–431)
**BODE Index**	N/A	N/A	5 (1–6)	5 (2–7)

Results are expressed as median with range in brackets.

**Table 3:** Demographic and spirometric values of the induced sputum studied groups.

	Healthy non-smokers	Healthy smokers	exS-COPD	cuS-COPD
**Participants**	5	10	6	5
**Age (years)**	51 (42–68)	51 (44–68)	62 (44–69)	69 (61–73)
**Gender (M/F)**	1/4	5/5	4/2	2/3
**Packs/yrs**	0 (0)	35 (19–51)	49 (27–77)	51 (41–62)
**Smoking status (Current/Ex)**	0	10/0	0/6	5/0
**Chronic bronchitis** **(Yes/No)**	0	4/6	3/3	4/1
**FEV_1_ (% pred)**	112 (88–124)	100 (89–122)	59 (37–73)	59 (45–68)
**FEV_1_/FVC (%)**	78 (72–86)	74 (73–82)	55 (39–69)	45 (39–59)
**▵FEV_1_ post bronch**	2.2 (1.1–3.5)	1.6 (1.1–2.7)	3.8 (3.6–4.1)	3.9 (3.6–4.3)
**BMI (kg/m^2^)**	26.4 (18.9–29.3)	23.8 (20.0–31.0)	27.2 (20.2–34.0)	23.5 (19.3–25.7)
**Inhaled GCS (on/off)**	N/A	N/A	4/2	3/2
**MRC dyspnoea scale**	N/A	N/A	3 (2–4)	3 (2–4)
**Distance walked in 6 min (m)**	N/A	N/A	359 (168–554)	288 (225–390)
**BODE Index**	N/A	N/A	4 (1–7)	5 (2–6)

Results are expressed as median with range in brackets.

### Peripheral Blood Mononuclear Cell isolation

Peripheral blood mononuclear cells (PBMCs) were isolated from whole blood on a discontinuous histopaque density gradient (Sigma, Poole, UK). Briefly, 50 ml of whole blood were diluted 2∶1 in RPMI 1640 medium (Sigma, Poole, UK), layered over histopaque and centrifuged for 22 minutes at 800 g. The resultant mononuclear layer was removed and washed twice and resuspended.

### Sputum induction and cell isolation

Sputum was induced by inhalation of hypertonic saline as described previously [31] with the difference that the participant was given salbutamol (400 mg) via a volumatic, rather than 1 mg terbutaline. The sample was placed on ice and processed within 1 hour. The cells were isolated as previously described [30]. Briefly, sputum was mixed with an equal volume of PBS (Sigma, Poole, UK), then filtered through 48 µm nylon gauze (Sefar, Bury, UK). An aliquot was taken for cell counting to assess viability and squamous cell contamination, and the remaining sample was resuspended for use in flow cytometry.

### Flow cytometric analysis

Cells were stained for flow cytometry as previously described [29]. Briefly, cells were fixed in 3% formaldehyde in isotonic azide free solution (Beckman Coulter, Luton, UK) and washed before labelled antibodies (Table 4) were added at the recommended concentration. The cells were incubated in the dark and excess antibody removed. Flow cytometric analysis of antibody labelled cells was performed using an EPICS Altra (Beckman Coulter, Luton, UK). Fifty thousand live-gated events were collected for each sample and isotype matched antibodies were used to determine binding specificity. Data were analysed using WEASEL version 2.3 (WEHI). Necrotic cells were excluded from analysis according to their forward and side scatter characteristics.

**Table 4:** Antibodies used for flow cytometry.

Antigen	Fluorochrome	Isotype	Clone	Source
CD3	ECDPC7	Mouse IgG1	UCHT1	Beckman Coulter, Luton, UK
CD8	PC5APC	Mouse IgG1	B9.11	Beckman Coulter, Luton, UK
CD8	ECD	Mouse IgG1	SFCl21Thy2D3	Beckman Coulter, Luton, UK
CD25	FITCPE	Mouse IgG2a, κ	B1.49.9	Beckman Coulter, Luton, UK
CD56	PEPC5PC7	Mouse IgG1	N901	Beckman Coulter, Luton, UK
CD69	ECDPC5	Mouse IgG2b	TP1.55.3	Beckman Coulter, Luton, UK
KIR3DL1(CD158e1)	FITC	Mouse IgG1, κ	DX9	Biolegend, San Diego, CA, USA
NKG2D (CD314)	PE	Mouse IgG1	ON72	Beckman Coulter, Luton, UK

### Statistical analysis

The statistical analysis was performed with Prism software, version 4.0c (GraphPad). Normality was detected using the Kolmogorov–Smirnov test. As some data were non-normally distributed all are expressed as median and range, unless otherwise stated. Differences between the three groups of participants were tested using the non-parametric Kruskal-Wallis test with *post hoc* pairwise comparisons made by the Dunn's Multiple Comparison test to determine which pair was statistically significantly different. P values ≤0.05 were considered to indicate statistical significance.

## Results

### Demographic and Medical Characteristics of COPD participants, Healthy Smokers and Healthy Controls

There were no statistical differences between groups in terms of age or pack years smoked between ex-smokers with COPD, current smokers with COPD and healthy smokers. Furthermore, there was no statistical difference in the inhaled corticosteroid use between ex-smokers with COPD and current smokers with COPD.

### Killer cell activation in peripheral blood

**Figure 1:**
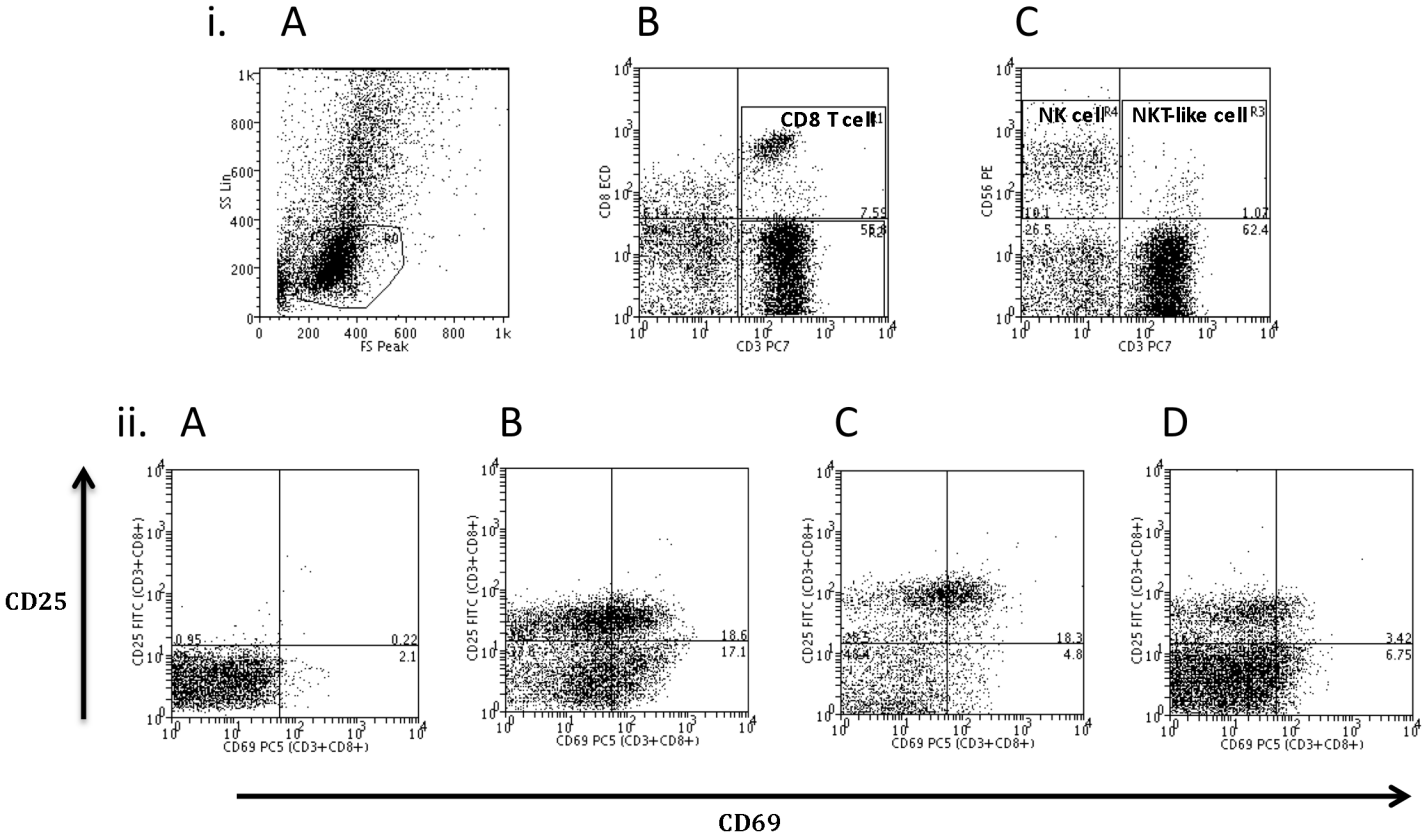
Representative dot plots gating for live/dead cells, identification of cell populations and expression of activation markers by CD8^+^ T lymphocytes from four groups. (i)(A) Forward Scatter (FS) vs Side Scatter (SS) plot for identification of live and dead cells; (B) Identification of CD8^+^ T lymphocytes (CD3+CD8+); and (C) identification of NK cells (CD3-CD56+) and NKT-like cells (CD3+CD56+). (ii) Representative dot plots for activation of CD8+ T lymphocytes expressing either CD69 alone (lower right quadrant), CD69 and CD25 (upper right quadrant) or CD25 alone (upper left quadrant) in four groups: (A) Healthy non-smoking participant; (B) Healthy smoker; (C) Current smoker with COPD; and (D) ex-smoker with COPD.

**Figure 2:**
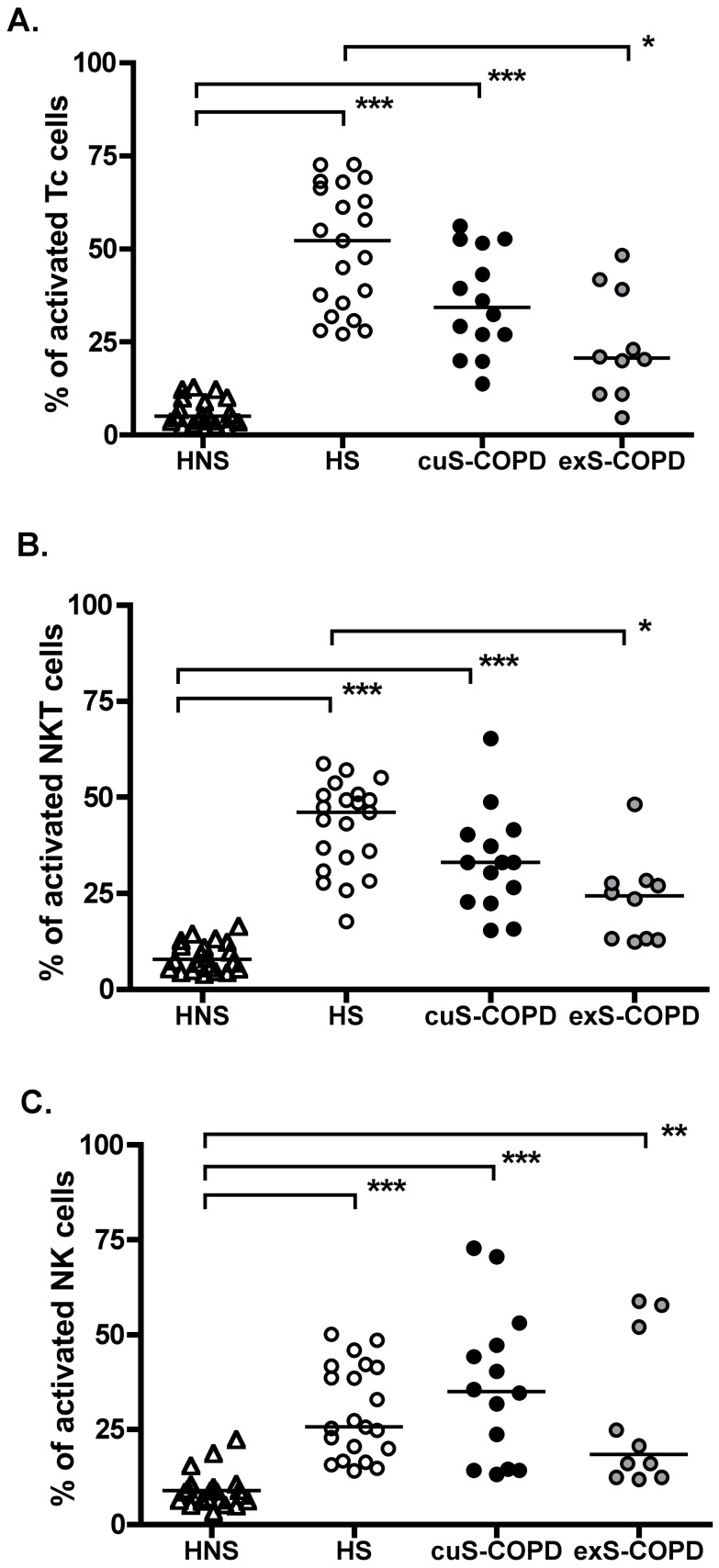
Activation of killer cells from peripheral blood *ex vivo* of four groups. Activation of CD8^+^ T-lymphocytes, NKT-like cells and NK cells was analysed. There was a significant increase in level of activation *ex vivo* of CD8^+^ T lymphocytes (panel A), NKT-like (CD56^+^CD3^+^) cells (panel B) and NK (CD56^+^CD3^−^) cells (panel C) from both healthy smokers (HS, open circles) and cuS-COPD (solid circles). Activation of NK (CD56^+^CD3^−^) cells from exS-COPD (grey solid circles) was also significantly increased compared with healthy non-smokers (HNS). *: p<0.05, **: p<0.01, ***: p<0.001. HNS (n = 21), HS (n = 21), cuS-COPD participants (n = 14) and exS-COPD (n = 10).

All individuals had similar total mononuclear cell numbers, which were within the normal range. Representative flow analysis dot plots of Forward Scatter (FS) vs Side Scatter (SS) for identification of live and dead cells, together with dot plots for identification of CD8^+^ T-lymphocytes (CD3+CD8+); NK cells (CD3-CD56+) and NKT-like cells (CD3+CD56+), along with dot plots for CD69 and CD25 staining of peripheral blood CD8^+^ T-lymphocytes, are shown in Figure 1. Cells are designated activated when they express one or both of the activation markers (i.e., CD69 positive, CD69/CD25 double positive or CD25 positive). Significantly more CD8^+^ T-lymphocytes from both healthy smokers (52.3%, 27.2–72.8; p<0.001) and current smokers with COPD (34.3%, 13.8–56.2; p<0.001) were activated *ex vivo* compared to healthy non-smokers (5.1%, 2.5–13.3) (Figure 2A). However, there were no significant differences in the activation of CD8^+^ T-lymphocytes between ex-smokers (24.1%, 4.7–48.4) and healthy smokers (52.3%, 27.2–72.8; p>0.05) or current smokers with COPD (34.3%, 13.8–56.2; p>0.05). Analyses of individual markers (i.e., CD69 positive/CD25 negative; CD69 negative/CD25 positive; OR CD69 positive/CD25 positive) were not different between groups or cell types (data not shown).

NKT-like cells showed a similar pattern to CD8^+^ T-lymphocytes, namely significantly more *ex vivo* activation from both healthy smokers (46.0%, 17.7–58.7; p<0.001) and current smokers with COPD (33.0%, 15.4–65.3; p<0.001) compared to healthy non-smokers (7.8%, 4.3–16.9) (Figure 2B). Also, no significant differences in NKT-like cell activation were observed between ex-smokers (24.3%, 12.4–48.1) and healthy non-smoker (7.8%, 4.3–16.9; p>0.05) or current smokers with COPD (33.0%, 15.4–65.3; p>0.05).

NK (CD56^+^CD3^−^) cells from healthy smokers (25.6%, 14.1–50.1; p<0.001), current smokers with COPD (35.1%, 13.2–72.8; p<0.001) and ex-smokers with COPD (18.4%, 11.9–58.8; p<0.005) were significantly more activated *ex vivo* than those from healthy non-smokers (8.9%, 3.6–22.8) (Figure 2C).

**Figure 3:**
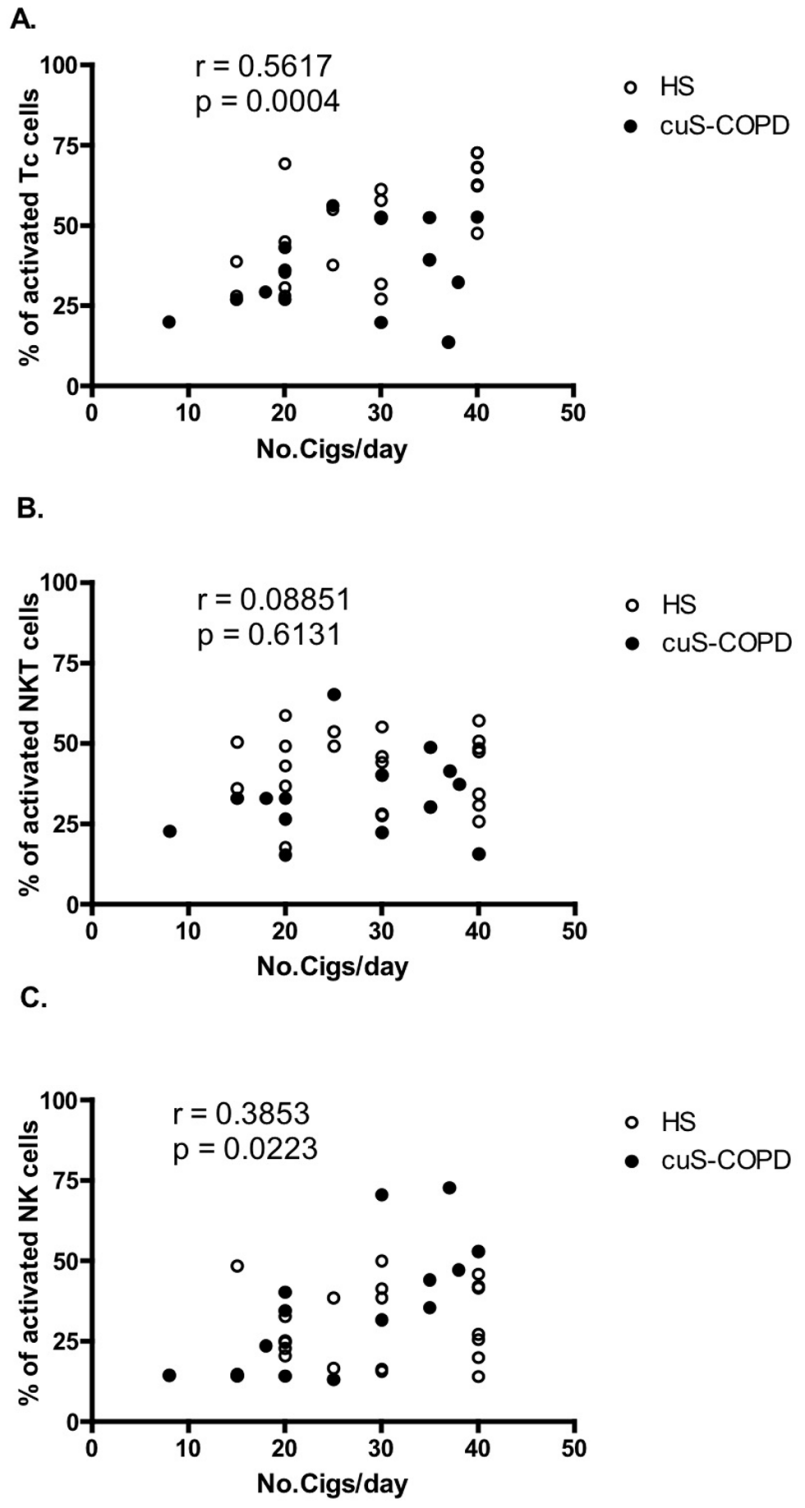
Correlation of the proportion of cells activated and number of cigarettes currently smoked per day. Analysis was performed on peripheral blood CD8^+^ T lymphocytes (Panel A), NKT-like (CD56^+^CD3^+^) cells (Panel B) and NK (CD56^+^CD3^−^) cells (Panel C). A significant correlation was observed in CD8^+^ T lymphocytes and NK (CD56^+^CD3^−^) cells but not NKT-like (CD56^+^CD3^+^) cells.

In view of our findings of the effect of current smoking we looked for correlations between the number of cigarettes currently being smoked per day and level of *ex vivo* cell activation for the three cell types (Figure 3). CD8^+^ T-lymphocytes and NK cells showed a significant positive correlation between the proportion of *ex vivo* activation and number of cigarettes smoked (r = 0.5617, p = 0.0004; r = 0.3853, p = 0.0223, respectively) (Figures 3A and 3C). No such correlation was observed for NKT-like cells (r = 0.08851, p = 0.6131) (Figure 3B).

### CD158e1 and NKG2D expression in peripheral blood

**Figure 4:**
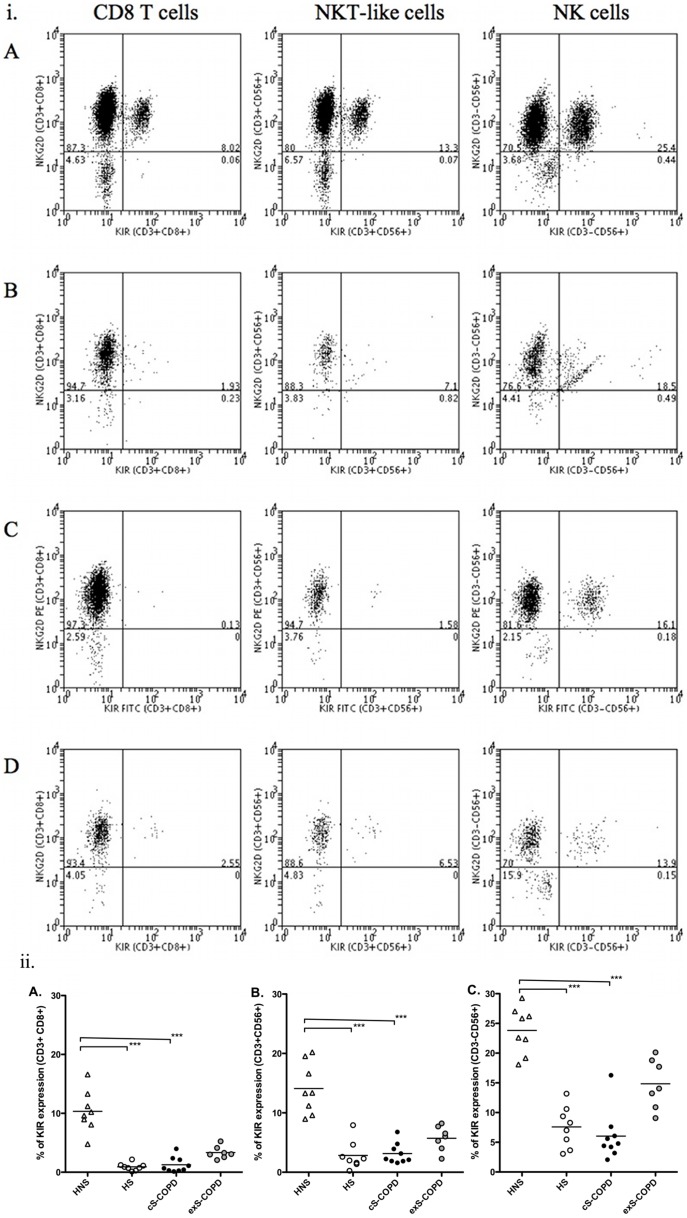
Expression of KIR by peripheral blood cells in four groups. (i) Representative dot plots of KIR expression: (Panel A) healthy non-smokers; (Panel B) healthy smokers; (Panel C) current smokers with COPD and (Panel D) ex-smokers with COPD. (ii) CD8^+^ T lymphocytes (Panel A), NKT-like (CD56^+^CD3^+^) cells (Panel B) and NK (CD56^+^CD3^−^) cells (Panel C). A significantly lower proportion of CD8^+^ T lymphocytes, NKT-like (CD56^+^CD3^+^) cells and NK (CD56^+^CD3^−^) cells from healthy smokers (HS, open circles) and cuS-COPD (solid circles) expressed KIR on the cell surface compared to healthy non-smokers (HNS). ***: p<0.001. HNS (n = 8); HS (n = 8); cuS-COPD participants (n = 9) and exS-COPD (n = 7).

To examine a possible effect of cell activation on the function of killer cells, expression of the inhibitory KIR CD158e1 was measured by flow cytometry (Figure 4). Representative dot plots of KIR expression from healthy non-smokers (Panel A); healthy smokers (Panel B); current smokers with COPD (Panel C) and ex-smokers with COPD (Panel D) are shown in Figure 4i.

A significantly lower proportion of CD8^+^ T-lymphocytes from healthy smokers (0.8%, 0.1–2.2; p<0.001) and current smokers with COPD (0.7%, 0.1–3.9; p<0.001) expressed CD158e1 on the cell surface compared to healthy non-smokers (9.9%, 4.8–16.6) (Figure 4iiA).

The proportion of NKT-like cells expressing surface CD158e1 was also significantly lower in healthy smokers (2.1%, 0.2–7.9; p<0.001) and current smokers with COPD (2.2%, 1.6–6.8; p<0.001) compared to healthy non-smokers (13.3%, 9.0–20.2) (Figure 4iiB).

Analysis of NK cells showed the proportion of these cells expressing surface CD158e1 was also significantly lower in healthy smokers (7.7%, 3.1–13.2; p<0.01) and current smokers with COPD (4.8%, 2.1–16.3; p<0.001) compared to healthy non-smokers (24.2%, 18.1–29.3) (Figure 4iiC).

No differences in proportions of CD8^+^ T-lymphocytes, NKT-like cells or NK cells (in all cases over 95%) expressing NKG2D were shown between the four groups (data not shown).

### Killer cell activation in induced sputum

**Figure 5:**
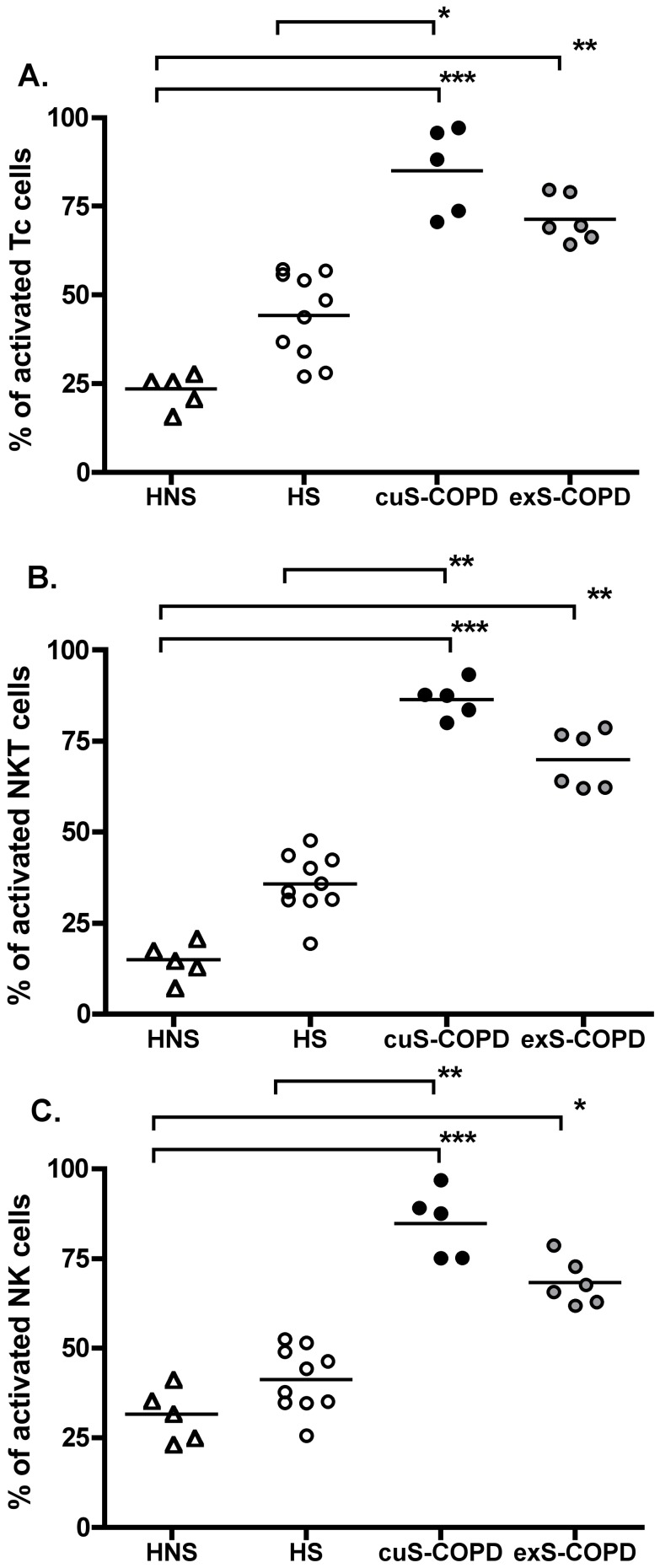
Activation of killer cells in induced sputum of four groups. CD8^+^ T-lymphocytes (Panel A), NKT-like (CD56^+^CD3^+^) cells (Panel B) and NK (CD56^+^CD3^−^) cells (Panel C). A significant increase in level of activation ex vivo of CD8^+^ T lymphocytes, NKT-like (CD56^+^CD3^+^) cells and NK (CD56^+^CD3^−^) cells from COPD patients with or without smoking history compared with healthy non-smokers (HNS). *: p<0.05, **: p<0.01, ***: p<0.001. HNS (n = 5), HS (n = 10), cuS-COPD participants (n = 5) and exS-COPD (n = 6).

In contrast to peripheral blood, flow cytometric analysis of the killer cells from induced sputum showed no effect of current smoking *per se* (Figure 5). Specifically, a significantly higher proportion of CD8^+^ T-lymphocytes from both current smokers with COPD (88.2%, 70.6–97.1; p<0.001) and ex-smokers with COPD (69.3%, 64.3–79.7; p<0.005) were activated *ex vivo* compared to healthy non-smokers (26.1%, 16.2–28.2). Furthermore, a significantly higher proportion of CD8^+^ T-lymphocytes from current smokers with COPD (88.2%, 70.6–97.1; p<0.05) were activated compared to healthy smokers (46.2%, 27.0–57.3) (Figure 5A).

The proportion of activated NKT-like cells *ex vivo* was significantly higher from both current smokers with COPD (87.5%, 80.0–93.3; p<0.001) and ex-smokers with COPD (69.8%, 62.0–78.7; p<0.005) compared to healthy non-smokers (15.0%, 7.5–21.0). In addition, a significantly higher proportion of NKT-like (CD56^+^CD3^+^) cells from current smokers with COPD (87.5%, 80.0–93.3; p<0.005,) were activated compared to healthy smokers (34.8%, 19.4–47.7) (Figure 5B).

The proportion of activated NK cells *ex vivo* was significantly higher from both current smokers with COPD (87.5%, 75.1–96.9; p<0.001) and ex-smokers with COPD (66.7%, 61.9–78.7; p<0.05) compared to healthy non-smokers (32.1%, 23.5–41.6). In addition, a significantly higher proportion of NK (CD56^+^CD3^−^) cells from current smokers with COPD (87.5%, 75.1–96.9; p<0.005) were activated compared to healthy smokers (41.1%, 25.6–52.5) (Figure 5C).

There was no difference in cell viability (% total) in induced sputum between current and ex-smokers with COPD, healthy smokers and healthy non-smokers (median, range): 83% (75–92), 84% (79–94), 82% (72–93) and 86% (75–94), respectively. In all samples, more than 90% of the cells were non-squamous.

## Discussion

Here we report, for the first time, that current smoking *per se* is associated with a significantly higher proportion of peripheral blood cytotoxic cells that are activated *ex vivo* and this is positively correlated with the number of cigarettes currently smoked per day. Furthermore, we show that, in induced sputum, it is the presence of COPD, rather than current smoking, that relates to cytotoxic cell activation.

We have previously demonstrated the potential involvement of the three main classes of human killer cells, namely, CD8^+^ T-lymphocytes, NKT-like cells and NK cells in COPD (7, 10, 11). These killer cells play key roles in inflammatory responses and activation of these cells can cause the production of inflammatory cytokines and chemokines, such as TNF-α and IL-8, that can induce pathological features of COPD and lung tissue damage [32]. Therefore, we hypothesised that killer cells are more active in COPD patients. Here we examined the *ex vivo* activation phenotype of these cells both in the peripheral blood and induced sputum. In peripheral blood, current smokers with or without COPD have the highest proportion of activated CD8^+^ T-lymphocytes, NKT-like cells and NK cells compared with healthy non-smokers and ex-smokers with COPD. This suggests that smoking *per se* affects systemic killer cell activation. We thus examined the correlation between current smoking profiles and cell activation levels. Here we show that CD8^+^ T-lymphocyte and NK cell activation is positively correlated with the number of cigarettes currently smoked. No correlations between cell activation and lung function (FEV_1_ percentage of predicted) were found (data not shown). These data suggest that, in peripheral blood, cell activation levels are more related to current smoking habit rather than disease.

Similar studies have shown that ex-smokers with COPD have significantly fewer CD8^+^CD25^+^ T-lymphocytes in BAL fluid compared to current smokers with COPD [28]. Furthermore, examining systemic CD4^+^ T-lymphocytes, also thought to be important in the pathogenesis of COPD, have shown a correlation between CD69 expression and lung function in COPD [26]. Sputum T-lymphocytes have been shown to express higher levels of CD103 and CD69 than blood lymphocytes, suggesting higher numbers of activated intra-epithelial phenotype T-lymphocytes in the lung [27].

To investigate a possible mechanism for differences in activation states observed in peripheral blood, we compared expression of the KIR CD158e1. Interestingly, in our study, the expression levels of the inhibitory KIR CD158e1 were lower in peripheral blood of current smokers with or without COPD compared to healthy non-smokers, which is the opposite to the activation levels of these killer cells. This suggests that the down regulation of the inhibitory KIRs may be associated with cell activation. There is evidence that expression of KIR may be a feature of differentiation towards a memory phenotype [33]. However, these cells possess not only inhibitory receptors but also activating receptors and killing ability is determined by a balance of signals from both types of receptors. We also compared the expression levels of one activating receptor NKG2D. Unlike CD158e1 KIR expression, no significant differences in NKG2D expression were measured between the four groups. They were all highly expressed by those activated cells (data not shown). This further supports the proposal of increased peripheral blood killer cell activity in smokers due to the increased ratio of activating to inhibitory receptors.

In contrast to our findings in peripheral blood, in induced sputum the proportion of activated cells is more related to disease state than current smoking, with both current and ex-smokers with COPD having significantly higher rates of activation than healthy non-smokers and healthy smokers. One hypothesis for these differences is that, in COPD, activated killer cells may have migrated to the lung from peripheral blood. Due to size differences between these compartments it is not surprising that this increased migration does not significantly affect the proportion of cells within the blood. A previous study examining the effect of smoking cessation has shown in BAL fluid that CD8^+^ T-lymphocytes from both smokers with normal lung function and COPD participants expressed high levels of CD69 and CD25 activation markers [28]. This difference may represent the use of lavage rather than induced sputum used in our study.

The present study has some limitations that deserve comment. Firstly, we used CD69 and CD25 as activation markers. Although other markers exist, these are generally accepted as representing cell activation [34], [35]. Secondly, CD25 is commonly used as a marker for T regulatory cells but these cells will express high levels of CD25 and for the present study only moderate levels were detected [36]. Thirdly, there are many kinds of inhibitory and activating receptors expressed on these cells but, due to limited cell numbers, we analysed two representative receptors that give an indication of the killing function of these cells. Fourthly, it should be noted that the majority of participants with COPD were taking inhaled corticosteroids. However, the two participants who were not on inhaled corticosteroids in this group showed the same results in terms of activation and KIR expression as those on inhaled corticosteroids. Finally, we used induced sputum to examine the activation state of cells. This does not reflect changes in the lower airway or within bronchial tissue but it is a convenient and non-invasive research tool [37], [38]. It would be important to follow up our findings by investigating BAL and bronchial biopsies in these groups of patients.

### Conclusion

In summary, we have shown, for the first time, a differential effect in systemic activation and lung activation of killer cells in COPD. Importantly, we have shown that systemic activation is in response to smoking *per se* whereas lung activation is due specifically to COPD, irrespective of current smoking status. Taken together, these findings indicate that activation of killer cells within the airways of COPD participants may play a role in the pathogenesis of COPD.
